# Zenker’s diverticulitis: a bitter pill to swallow

**DOI:** 10.1093/jscr/rjac258

**Published:** 2022-06-30

**Authors:** R J O’Neill, E F Cleere, N Elsafty, R Gaffney

**Affiliations:** Beaumont Hospital, Dublin, Ireland; Royal College of Surgeons, Dublin, Ireland; Beaumont Hospital, Dublin, Ireland; Beaumont Hospital, Dublin, Ireland; Royal College of Surgeons, Dublin, Ireland; Beaumont Hospital, Dublin, Ireland

**Keywords:** oesophageal obstruction, pharyngeal pouch, Zenker’s diverticulum

## Abstract

Acute oesophageal obstruction from food bolus impaction is often triggered by underlying oesophageal pathology, both benign and malignant. These can be readily detected with standard investigations such as oesophagoscopy or computed tomography. Zenker’s diverticulum (ZD) is a benign condition frequently presenting with chronic dysphagia or may be asymptomatic. We report the case of an 81-year-old man with a previously undiagnosed 1-cm ZD causing complete oesophageal obstruction secondary to localized oedema from an impacted ibuprofen tablet. Although initial clinical, endoscopic and radiological findings were equivocal and suspicious for upper oesophageal malignancy, symptoms rapidly settled in response to systemic corticosteroids. The diagnosis was later confirmed on barium swallow with no other clinical, radiological or histopathological abnormalities identified. In conclusion, ZD is an uncommon cause of acute oesophageal obstruction which may occur in diverticula of all sizes. Surgery should be performed in patients with recurrent symptoms or large diverticula.

## INTRODUCTION

Acute oesophageal obstruction is a common presenting complaint to the Emergency Department, where it is often secondary to poorly masticated food (often termed ‘Steakhouse Syndrome’) [1]. There is often underlying pathology, especially in recurrent or refractory cases, of which the differential includes benign (e.g. Schatzki ring) and malignant (e.g. oesophageal tumour) aetiologies [2]. Many of these are often readily identified on endoscopic examination. Zenker’s diverticulum (ZD) is a rare cause of acute oesophageal obstruction which is frequently overlooked [2, 3]. ZD more frequently presents with chronic symptoms, such as dysphagia, globus and halitosis, but small diverticula are often asymptomatic. We present a case of acute oesophageal obstruction secondary to tablet impaction in a small ZD in a previously asymptomatic and undiagnosed patient.

## CASE REPORT

An 81-year-old man presented to our Emergency Department with a 4-hour history of acute onset aphagia which developed after ingestion of two ibuprofen tablets that the patient felt stuck in his throat. There were no antecedent symptoms such as odynophagia, dysphagia or dysphonia. There was no history of large food bolus or bone ingestion in the preceding 24 hours. His past medical history was significant for vascular dementia, chronic obstructive pulmonary disease and peripheral vascular disease.

**Figure 1:**
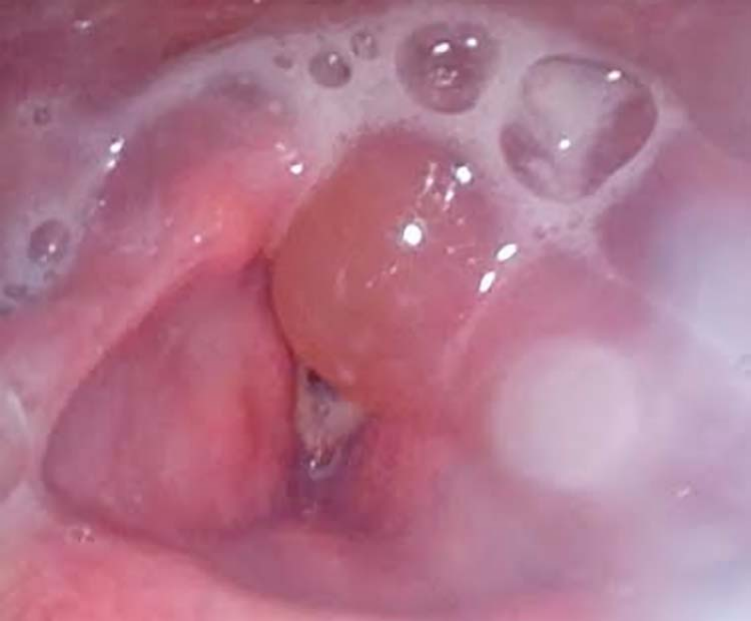
Laryngoscopy findings.

On review, the patient was agitated, drooling and unable to swallow oral secretions. He was otherwise systemically well with no features of airway compromise. He was initially treated with hyoscine butylbromide (Buscopan®) and glucagon with no symptomatic improvement. Serum parameters, including haemoglobin and C-reactive protein, were normal. White cell count was mildly elevated (12.4 × 10^9^/l). Flexible laryngoscopy revealed laryngeal pooling of secretions and gross oedema of the left aryepiglottic fold, piriform fossa and post-cricoid space (Fig. 1). Following this, dexamethasone was commenced.

**Figure 2:**
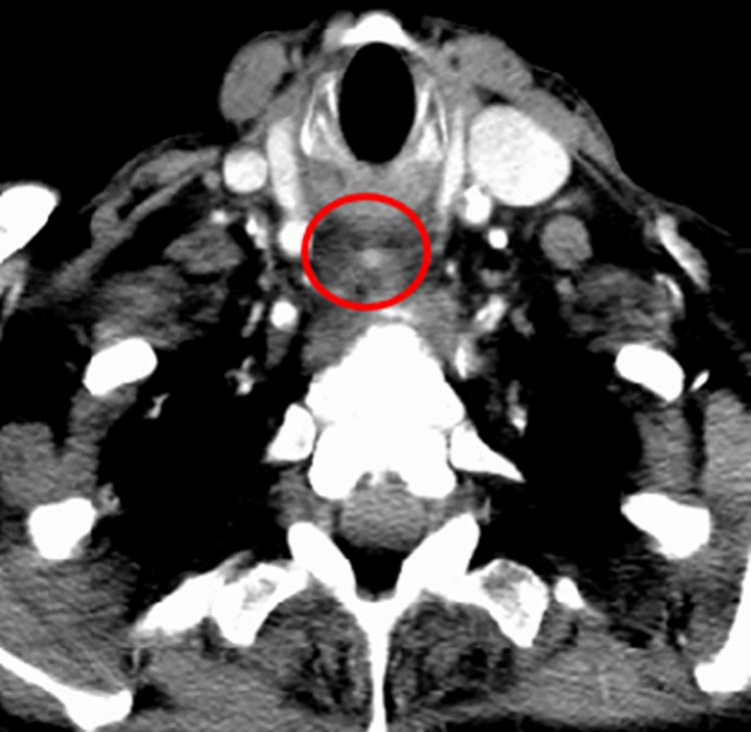
CT neck; post-cricoid mass containing an air locule with a 3-mm hyperdense mass (red circle) in the caudal end of this.

Computed tomography (CT) was performed showing a large, ill-defined mass of which the exact aetiology was initially unclear but suspicious for a primary hypopharyngeal malignancy. Of note, within this mass, a small air collection was observed, containing a 3-mm hyperdense focus (Fig. 2).

The patient experienced a rapid symptomatic improvement in response to dexamethasone. Rigid oesophagoscopy and direct laryngoscopy were performed the following morning, which showed near total resolution of the supraglottic oedema. Clinically, there were no features suggestive of malignancy in the hypopharynx or upper oesophagus. However, a small dimple in the post-cricoid area was noted. Supraglottic biopsies were taken for completion. Oral feeding was gradually recommenced.

**Figure 3:**
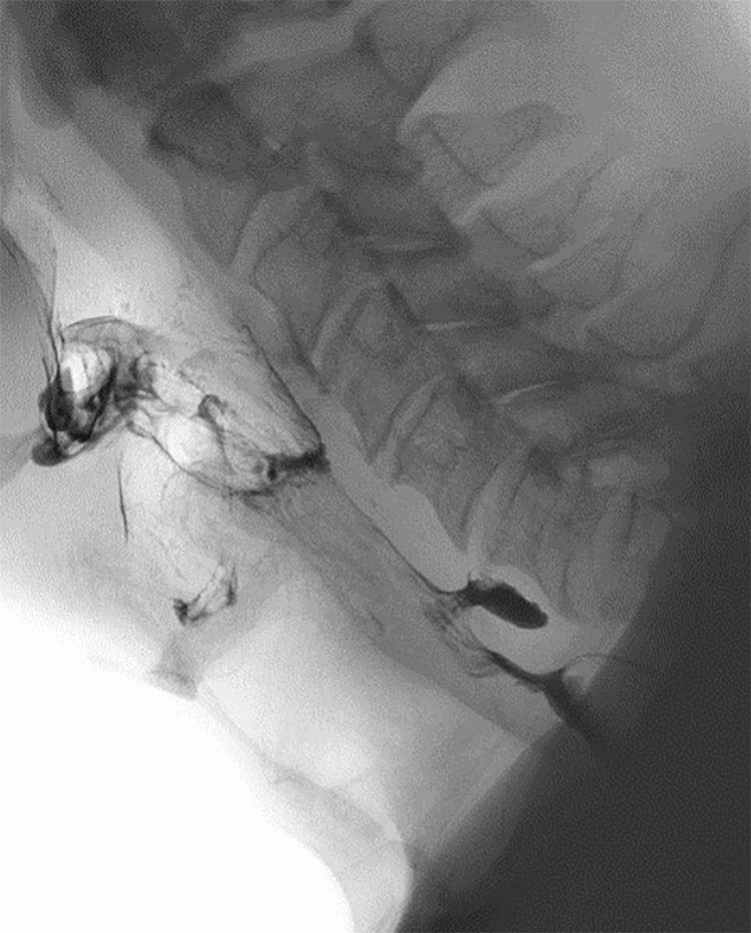
Barium swallow demonstrating 1.1-cm ZD.

Clinical and radiological findings were reviewed in a multidisciplinary setting where features were suspicious for ZD as an underlying aetiology. This was confirmed on barium swallow which demonstrated a 1.1-cm ZD (Fig. 3).

The patient was discharged after one week and reviewed six weeks post-operatively where repeat flexible laryngoscopy was normal. He remained clinically well and was grossly asymptomatic. Histopathology was benign and only demonstrated inflammatory squamous mucosa. Given the small diverticulum size, patient age, co-morbidities and lack of recurrent symptoms, the patient was managed with clinical observation with no recurrence to date.

## DISCUSSION

ZD, also known as pharyngeal pouch, is an outpouching of pharyngeal mucosa through Killian’s triangle, an anatomical area of weakness between the cricopharyngeus and thyropharyngeus muscles [4]. They are more common in the elderly with an estimated incidence of 2/100 000 [2, 5]. Symptoms are typically chronic and include globus, dysphagia and halitosis. Less common presentations include neck mass, recurrent aspiration pneumonia and dysphonia [6]. However, ZD are frequently asymptomatic. Squamous cell carcinoma is a rare complication, occurring in ~1% of cases [3]. Acute presentations are rare; only a handful of cases have been reported in the last 20 years which are summarized in Table 1 [3, 5, 7–9].

**Table 1:** Cases of acute oesophageal obstruction secondary to ZD

Case	History	Management	Outcome
Ney, 2019	78-year old man. Acute obstruction with mild haematemesis. Food bolus impacting large ZD	OGD followed by evacuation of bolus via rigid esophagosopy	Symptom resolution. Planned for elective surgery to prevent recurrence
Mantziari, 2018	78-year-old man. Known 8-cm ZD, progressive symptoms	OGD, open diverticulopexy, cricopharyngeal myotomy	No reported complications. Clinically well 4 weeks post-operatively
Carmel-Neiderman, 2017	Man aged >70, known 5-cm ZD, progressive symptoms, cachexia	Endoscopic stapling diverticulotomy	Asymptomatic 3 months post-operatively with weight gain
Leite, 2015	76-year-old man, progressive dysphagia, cachexia, 4-cm ZD	Witzel jejunostomy	Symptom resolution. Planned for elective surgical treatment
Geisler, 2002	86-year-old man, known 6-cm ZD, progressive dysphagia	Six-stage endoscopic diverticulotomy using argon plasma coagulation	Symptom resolution, weight gain

There are notable differences between our case and others previously reported. Although acute oesophageal obstruction in ZD has been previously described, it appears to occur exclusively in the presence of large diverticula (>4 cm) which are often impacted with food. Our patient had a small 1.1-cm ZD without food impaction. Identifying the presence of food bolus impaction is critical in these patients due to an increased risk of major complications, such as oesophageal ischemia, perforation or mediastinitis, if the impaction is not addressed within 24 hours of onset [1]. Our patient was previously asymptomatic and had no antecedent symptoms which is in contrast to other published cases. In the present case, it appeared that oesophageal obstruction was caused by localized oedema of the diverticulum triggered by the impaction of an ibuprofen tablet into this, which correlates with the small hyperdense area at the centre of the phlegmon observed on CT (Fig. 2). This is supported by the patient’s history, laryngoscopy findings, imaging and rapid response to intravenous steroids. Due to this, ZD may be an appropriate term. Oral medications, especially ibuprofen, causing oesophageal oedema and obstruction have been described, however, this is usually caused by the large tablet size [10].

Initially the aetiology was unclear: imaging and endoscopic findings were equivocal and the patient age and smoking history raised concern for underlying malignancy. However, it was the multidisciplinary review that was important in identifying the final diagnosis. This case highlights the relevance of multidisciplinary input in such cases.

This patient was managed conservatively, which varies from other similar cases which seem to be managed more invasively likely due to chronic and progressive symptoms and generally larger ZD size (Table 1). There are a variety of surgical approaches for ZD, including endoscopic staple diverticulotomy and open transcervical diverticulum excision [11]. The choice of operation often depends on ZD size and surgeon experience [12]. ZD < 1 cm can often be managed conservatively. Surgical intervention is not without risk, especially in an elderly population. There is a modest recurrence rate [11].

## CONCLUSIONS

Clinicians should be aware of ZD as a cause of acute oesophageal obstruction. Correlation of clinical and radiological findings is essential. Food bolus impaction must primarily be excluded in these patients. Small ZD may be observed, but surgery should be considered for patients with recurrent symptoms or large diverticula.
